# Supplementary material for: The mitochondria-targeted derivative of the classical uncoupler of oxidative phosphorylation carbonyl cyanide m-chlorophenylhydrazone is an effective mitochondrial recoupler
Source: PLoS One. 2020 Dec 30;15(12):e0244499. doi: 10.1371/journal.pone.0244499 (PMC7773232; doi:10.1371/journal.pone.0244499)
Supplement: S1 File — (DOC) [file pone.0244499.s001.doc]

**Electronic Supplementary Information (ESI)**

The Mitochondria-targeted Derivative of the Classical Uncoupler of Oxidative Phosphorylation Carbonyl Cyanide *m*-Chlorophenylhydrazone is an Effective Mitochondrial Recoupler

Iliuza R. Iaubasarova,^1,2^ Ljudmila S. Khailova,^1^ Alexander M. Firsov,^1^ Vera G. [Grivennikova](https://istina.msu.ru/workers/478644/),^3^ Roman S. Kirsanov,^1^ Galina A. Korshunova,^1^ Elena A. Kotova,^1^ and Yuri N. Antonenko,^1^*

^1^Belozersky Institute of Physico-Chemical Biology, Lomonosov Moscow State University, Leninskie Gory 1/40, Moscow 119991, Russia;

^2^Faculty of Chemistry, Lomonosov Moscow State University, Leninskie Gory 1/3, Moscow 119991, Russia;

^c^Faculty of Biology, Lomonosov Moscow State University, Leninskie Gory 1/12, Moscow 119991, Russia;

*****Correspondence: [antonen@genebee.msu.ru](mailto:antonen@genebee.msu.ru) (Y.N.A.)

**Synthesis of 10-bromodecyl(triphenyl)phosphonium bromide**

A solution of 1,10-dibromodecane (3.0 g, 10 mmol) and triphenylphosphine (1.31 g, 5 mmol) in benzene (1 ml) was heated at 75°C during 24 hours in a tightly closed flask. After completing the reaction, the reaction mass was cooled at 20°C and transferred to a round-bottom flask with dichloromethane to evaporate to dryness. The residue obtained was dissolved in a minimal volume of dichloromethane, then an excess of hexane was added and the suspension formed was kept at 4°C until the solution became clear. Then the liquid phase was decanted, the residue was dissolved again in dichloromethane and treated with hexane to complete precipitation. This procedure was repeated three times. Finally, the residue was dissolved in a minimal volume of the methanol-dichloromethane (1:6) solvent system and applied to a chromatographic silica gel column (MN Kieselgel 60, 240-400 mesh) in the same solvent system as an eluent. Detection was carried out with the help of TLC by UV-absorbance and Dragendorff reaction. Fractions with the same chromatographic mobilities were combined and evaporated in vacuo (yield 2.4 g, 86 %)

LCMS: m/z: [M]^+^ found 483.3, required 483.4

**Synthesis of carbonyl cyanide p-hydroxy-m-chlorophenyl hydrazone**

4-Amino-2-chlorophenol (1.0 g, 7 mmol) was added to the aqueous hydrochloric acid solution (6.3 ml conc. HCl in 42 ml H_2_O). To this solution, pre-chilled with ice, a pre-chilled solution of sodium nitrite in 7 ml of water was added. The resulting solution was mixed in an ice bath for 2 hours. Then this solution was added with stirring to an aqueous solution of malononitrile (693 mg, 10.5 mmol) and sodium acetate (11.7 mg) in 70 ml of water. They were mixed in the cold for 30 minutes, then left in the refrigerator for the night. The yellow precipitate was filtered off and dissolved in a minimal amount of the chloroform-methanol (6:1) system. Then the mixture was separated on a silica gel column in the same system (yield 640 mg, 42 %).

TLC chloroform-methanol (6:1) R_f_ = 0.73

ESI-MS: m/z: [M-H]^-^ found 218.8, required 219.1

**Synthesis of 10-([4’-(dicyanomethylene)hydrazinyl-2’-chlorophenyl]oxy)decyl(triphenyl)phosphonium bromide (mitoCCCP)**

Сarbonyl cyanide *p*-hydroxy-m-chlorophenylhydrazone (51 mg, 0.23 mmol) and 10-bromodecyl(triphenyl)phosphonium bromide (239 mg, 0.43 mmol) were dissolved in 1.2 ml of absolute DMF. Cesium carbonate (114 mg, 0.35 mmol) and sodium iodide (24 mg) were added to the solution. The reaction was carried out at room temperature with stirring for 3.5 hours. After completion of the reaction, water was added to the reaction mixture in a 10-fold excess by volume. Then the aqueous solution was extracted with dichloromethane (30 ml, 3 times). The colored dichloromethane solution was dried with anhydrous sodium sulfate. Then dichloromethane was evaporated, the dry residue was dissolved in the chloroform-methanol (6:1) system and separated on a silica gel column in the same system (yield 20 mg, 12 %).

TLC chloroform-methanol (6:1) R_f_ = 0.59

ESI-MS: m/z: [M]^+^ found 621.8, required 621.3

**Fig.S1.** Electrospray ionization mass spectrum of carbonyl cyanide p-hydroxy-m-chlorophenyl hydrazone (*p-*hydroxyCCCP)


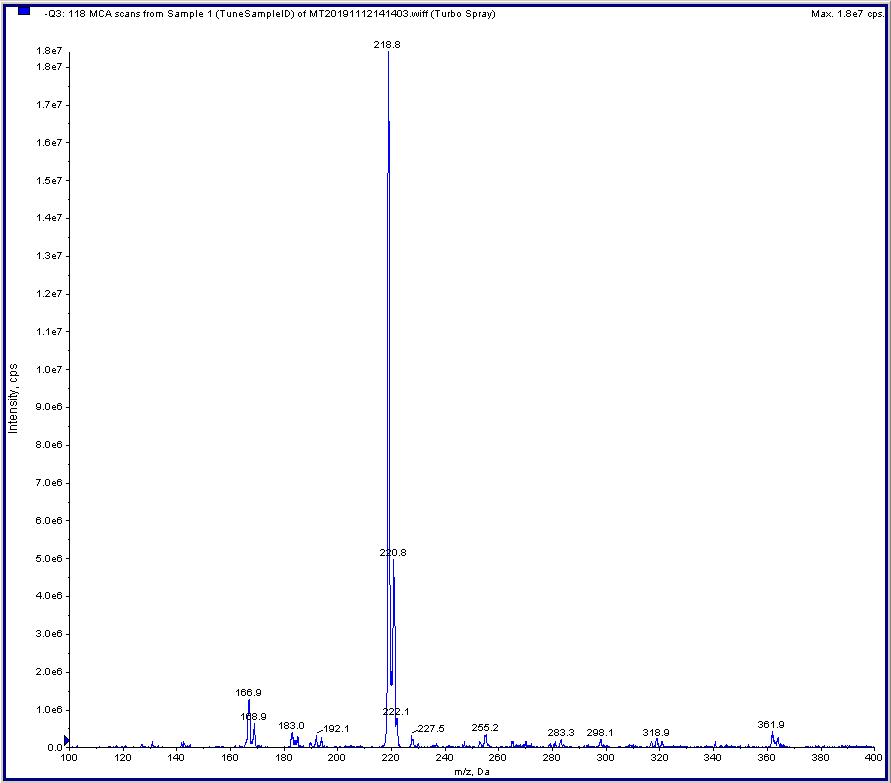

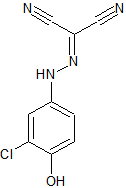


Empirical formula С_9_H_5_ClN_4_O

Calculated Molar Mass 220.62

ESI-MS: [M-H]^-^ 218.8 required 219.1

Mass spectrometric analysis parameters:

3200 Q TRAP LC / MS / MS System

The concentration of the compound (1-3) * 10 ^ (- 6)

Solvent Acetonitrile (LC / MS) - Water MQ (1: 1)

Ionization - Electrospray (ESI-MS)

IonSpray Voltage 4500V

Declustering Potential 40V

Entrance Potential 10V

**Fig.S2.** Electrospray ionization mass spectrum of


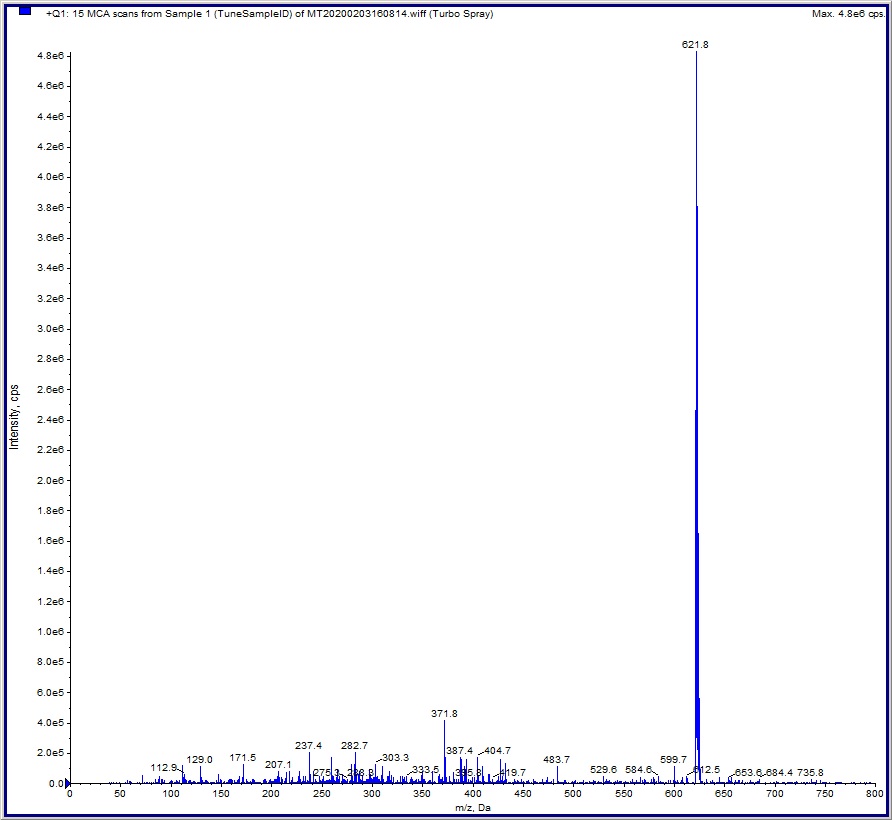

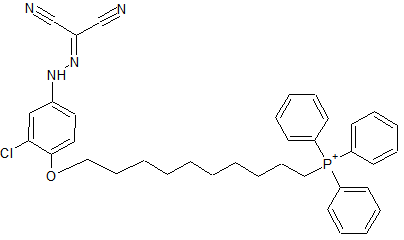


10-([4’-(dicyanomethylene)hydrazinyl-2’-chlorophenyl]oxy)decyl(triphenyl)phosphonium bromide (mitoCCCP)

Empirical formula С_37_H_39_ClN_4_OP

ESI-MS: [M]^+^ 621.8 required 621.3

Mass spectrometric analysis parameters:

3200 Q TRAP LC / MS / MS System

The concentration of the compound (1-3) * 10 ^ (- 6) M

Solvent Acetonitrile (LC / MS) - Water MQ (1: 1)

Ionization - Electrospray (ESI-MS)

IonSpray Voltage 4500V

Declustering Potential 80V

Entrance Potential 7V

**Concentration dependence of the recoupling effect of mitoCCCP on mitochondria uncoupled by CCCP**

Fig.S3 shows concentration dependence of the recoupling effect of mitoCCCP. The recoupling effect on the membrane potential was quantified one minute after the addition of mitoCCCP with RLM incubated with 200 nM CCCP as measured by changes in absorbance of the potential-sensitive dye safranine O.

**Fig. S3.** Dose dependence of the recoupling effect of mitoCCCP in the presence of 200 nM CCCP. Substrate: succinate. The membrane potential of mitochondria was estimated from changes in the absorbance of the potential- sensitive dye safranine O (15 µM) at 555 nm and 523 nm. The addition of 200 nM CCCP decreased the safranine signal to the level characteristic for high concentrations of uncouplers. Subsequent addition of mitoCCCP restored the signal. The initial signal without CCCP was set 100 %. Shown are Mean±S.D. (n=3). For other conditions, see Materials and Methods.

Fig.S4 shows the dependence of the recoupling effect of 2.5 µM mitoCCCP on the concentration of previously added CCCP. The recoupling action of mitoCCCP remained nearly complete in a wide range of CCCP concentrations except for concentrations exceeding 300 nM.

**Fig. S4.** Dose dependence of the recoupling effect of 2.5 µM mitoCCCP in the presence of various concentrations of CCCP added at t=60 s. Substrate: succinate. The membrane potential of mitochondria was estimated from changes in the absorbance of the potential sensitive dye safranine O (15 µM) at 555 nm and 523 nm. For other conditions, see Materials and Methods.

**Recoupling action of mitoCCCP in the presence of CCCP on rat liver mitoplasts**

Fig.S5 shows the recoupling action of mitoCCCP with mitoplasts. The addition of 100 nM CCCP caused stronger depolarization in mitoplasts compared to mitochondria (red and black curves). Subsequent addition of 3 µM mitoCCCP led to partial recoupling in the case of mitoplasts while the recoupling was nearly complete for mitochondria. Green curve in Fig.S5 shows the recoupling action of mitoCCCP in the case of a lower concentration of CCCP (50 nM). Interestingly the recoupling action of mitoCCCP exhibited transient character in the case of mitoplasts. The initial rise of the membrane potential after the addition of mitoCCCP was followed by a slow decrease lasting several minutes. It can be assumed that the slow disappearance of the effect of mitoCCCP in the case of mitoplasts could be accounted for by limited tightness of the inner mitochondrial membrane induced by a stage of high-amplitude swelling of mitochondria during the procedure of the preparation of mitoplasts.

**Fig.S5**. Effect of mitoCCCP (3 µM) on the uncoupling activity of CCCP in mitochondria (black curve) and mitoplasts (green and red curves). Substrate: succinate. The membrane potential of mitochondria was estimated from changes in the absorbance of the potential-sensitive dye safranine O (15 µM) at 555 nm and 523 nm. For other conditions, see Materials and methods.

**Spectrophotometric measurements of pKa of mitoCCCP**

Fig.S6 shows pH dependences of absorption spectra of mitoCCCP (A) in the presence of liposomes made from egg yolk phosphatidylcholine. Panel B shows the dependence of the wavelength of the absorbance maximum of mitoCCCP on pH. Black curve is the best fit by the Henderson-Hasselbalch equation with pK_a_=3.7.

**Fig.S6.** The pH dependences of absorption spectra of mitoCCCP (10 μM, A) in the presence of liposomes made from egg yolk phosphatidylcholine (25 μg/ml). The solution was 40 mM Tris, 40 mM MES, 40 mM beta-alanine, 10 mM KCl. **B**, The dependences of the wavelength of the absorbance maximum on pH. Black curve is the best fit by the Henderson-Hasselbalch equation with pK_a_=3.7.

**Effect of mitoCCCP and CCCP on subbacterial particles**

The pH gradient generated on subbacterial particles (SBP) of *E. coli* after the addition of succinate, manifesting itself in the quenching of ACMA fluorescence, was decreased by 60 nM mitoCCCP (Fig.S7, black curve), and the effect was similar to that of 200 nM CCCP (Fig.S7, red curve). The addition of 60 nM mitoCCCP after 200 nM CCCP led to an additional decrease in the pH gradient (red curve).

**Fig.S7.** Effect of mitoCCCP and CCCP on the pH gradient across the membranes of subbacterial particles (SBP), as measured by ACMA (0.5 µg/ml) fluorescence. Shown are traces of fluorescence at 480 nm (excited at 410 nm) in the medium containing 100 mM KCl, 10 mM HEPES, 5 mM MgCl_2_, pH 7.4 in the presence of 1.5 mM succinate. In the traces, SBP (25 µg/ml) was supplemented at t=200 s. 1 mM malonate was added at the end of each trace.
